# Tunable biphasic drug release from ethyl cellulose nanofibers fabricated using a modified coaxial electrospinning process

**DOI:** 10.1186/1556-276X-9-258

**Published:** 2014-05-23

**Authors:** Chen Li, Zhuan-Hua Wang, Deng-Guang Yu, Gareth R Williams

**Affiliations:** 1 Key Laboratory of Chemical Biology and Molecular Engineering, College of Life Science, Shanxi University, 92 Wucheng Road, Taiyuan 030006, China; 2 School of Materials Science & Engineering, University of Shanghai for Science and Technology, Shanghai 200093, China; 3 Upalitocacy, 29-39 Brunswick Square, London WC1N 1AX, UK

**Keywords:** Modified coaxial electrospinning, Core-shell nanofibers, Tunable biphasic release, PVC-coated spinneret, Ethyl cellulose

## Abstract

This manuscript reports a new type of drug-loaded core-shell nanofibers that provide tunable biphasic release of quercetin. The nanofibers were fabricated using a modified coaxial electrospinning process, in which a polyvinyl chloride (PVC)-coated concentric spinneret was employed. Poly (vinyl pyrrolidone) (PVP) and ethyl cellulose (EC) were used as the polymer matrices to form the shell and core parts of the nanofibers, respectively. Scanning and transmission electron microscopy demonstrated that the nanofibers had linear morphologies and core-shell structures. The quercetin was found to be present in the nanofibers in the amorphous physical status, on the basis of X-ray diffraction results. *In vitro* release profiles showed that the PVP shell very rapidly freed its drug cargo into the solution, while the EC core provided the succedent sustained release. Variation of the drug loading permitted the release profiles to be tuned.

## Background

Since the first report of drug-loaded nanofibers fabricated using electrospinning [1], these materials have been widely explored in the biomedical field [2-5]. As the electrospinning processes reported in the literature have become more complex, advancing from single-fluid to multiple-fluid processes [6-8], the nanofibers thereby produced have correspondingly evolved from monolithic nanofibers to core-shell structures, side-by-side nanofibers, and nanofibers containing particles or with a high porosity [9-11]. Current research is exploring how the electrospinning process could be scaled up from the laboratory to the industrial scale [12,13] and looking to improve the homogeneity and quality of the fiber populations generated [14,15]. Efforts are also underway to prepare increasingly complex nanofibers [8,16].

The most common way to generate drug-loaded nanofibers involves first preparing a co-dissolving solution of a drug and a carrier polymer, which is followed by electrospinning to remove the solvent [17]. Different types of release profile can be achieved by varying the polymer selected. For example, immediate drug release can be effected by preparing fibers of hydrophilic polymers such as poly (vinyl pyrrolidone) (PVP) and poly (ethylene oxide), while sustained release can be achieved with water-insoluble polymers or biodegradable macromolecules such as ethyl cellulose (EC), cellulose acetate, chitosan, and poly (ϵ-caprolactone) [15,18]. However, the possibilities are limited because there are only around 100 polymers that have good electrospinnability, and often electrospinning can only be achieved for particular molecular weights and within a very narrow concentration window [19]. Only around ten polymers have been used to prepare drug-loaded nanofibers and often the preparation conditions are extremely strict. Thus, the monolithic nanofibers which result from single-fluid electrospinning have limited applicability in the biomedical field. Coaxial electrospinning, employing a concentric spinneret with one needle nested inside another, has however been successfully employed to generate nanofibers from materials which cannot be electrospun in single-fluid processes [20]. Modified coaxial approaches, in which un-electrospinnable liquids are used as shell fluids with a core solution which has good electrospinnability, are further expanding the range of medicated nanofibers that can be fabricated [14,15].

Biphasic drug release profiles have drawn considerable attention in pharmaceutics for a number of reasons - one possible application is the ‘burst’ release of a loading dose of drug followed by sustained release over a prolonged period of time to maintain the systemic drug concentration within the therapeutic window [21-23]. A wide variety of technologies have been exploited to generate drug delivery systems with biphasic release profiles. Electrospinning can achieve this objective through strategies such as preparing multi-layered nanofiber mats or producing nanofibers containing nanoparticles [21,24]. Core-shell nanofibers generated using coaxial electrospinning have also been reported to offer biphasic release, with a fast-dissolving shell delivering immediate release followed by sustained release from the core [22]. Generally, both the core and shell fluids used for coaxial spinning have been electrospinnable in such studies [23].

Building on the developments discussed above, this study aimed to deliver three related goals: (i) the implementation of stable and effective coaxial electrospinning to generate high-quality core-shell nanofibers, (ii) employing modified coaxial electrospinning to prepare nanofibers using non-spinnable solutions, and (iii) manipulating structure-activity relationships at the nanoscale to yield accurate and adjustable time-programmed administration of drugs for specific therapeutic needs. A coaxial electrospinning process including a polyvinyl chloride (PVC)-coated concentric spinneret was implemented to prepare core-shell nanofibers of quercetin using an un-spinnable shell fluid containing PVP and quercetin. An electrospinnable solution of ethyl cellulose and quercetin was employed as the core fluid, and by controlling the concentration of quercetin in the shell fluid, the amount of drug released in the first phase of a biphasic drug release profile could be precisely tuned.

## Methods

### Materials

Quercetin (purity > 98%, No. MUST-12072505) was purchased from the Beijing Aoke Biological Technology Co. Ltd. (Beijing, China). PVP K30 (*M*_*w*_ = 58,000) was purchased from the Shanghai Yunhong Pharmaceutical Aids and Technology Co. Ltd. (Shanghai, China). EC (6 to 9 mPa s) was obtained from the Aladdin Chemistry Co. Ltd. (Shanghai, China). Methylene blue, *N*,*N*-dimethylacetamide (DMAc), and anhydrous ethanol were purchased from the Sinopharm Chemical Reagent Co. Ltd. (Shanghai, China). All other chemicals used were of analytical grade, and water was doubly distilled before use.

### Electrospinning

The core solutions were prepared by dissolving 24 g EC and 1 g quercetin in 100 mL of a solvent mixture comprising DMAc and ethanol in a volume ratio of 1:9. For initial optimization, an analogous solution was prepared, but quercetin was replaced by 2 mg of methylene blue. The shell solution was prepared by placing 35 g PVP and the desired amount of quercetin in 100 mL of a solvent mixture comprising DMAc and ethanol in a volume ratio of 3:7. Full details of the core solutions used are listed in Table 1. Initial optimization experiments were performed with shell solutions containing only PVP.

**Table 1:** Parameters of the electrospinning processes and their products

**Number**	**Process**	**Sheath drug content ( *w */ *v *) (%)**	**Flow rate (mL h**^**−1**^**)**		**Fiber morphology**^**c**^	**Diameter (nm)**
			**Sheath**^**a**^	**Core**^**b**^		
F1	Single	0	1.0	-	Film	-
F2		-	-	1.0	Linear	500 ± 180
F3	Coaxial	0	0.4	0.6	Mixed	-
F4		1.0	0.3	0.7	Linear	840 ± 140
F5		2.0		0.7	Linear	830 ± 140
F6		3.0		0.7	Linear	860 ± 120

^a^Sheath fluid consists of 35% (*w*/*v*) PVP K30 and different content of quercetin in a mixture of ethanol and DMAc with a volume ratio of 7:3. ^b^Core fluid consists of 20% (*w*/*v*) EC and 1% (*w*/*v*) of quercetin in a mixture of ethanol and DMAc with a volume ratio of 9:1. ^c^In this column, ‘linear’ morphology refers to nanofibers with few beads or spindles and ‘mixed’ morphology refers to linear nanofibers with beads.

A homemade PVC-coated concentric spinneret was prepared by inserting a metal concentric spinneret consisting of two stainless steel tubes (with inner diameters of 0.84 and 0.21 mm, respectively) into a PVC tube (inner diameter 1.0 mm, length 30 mm). The PVC tube projected 0.2 mm from the surface of the outer stainless steel tube and was even with the surface of the inner stainless steel tube. Two syringe pumps (KDS100 and KDS200, Cole-Parmer, Vernon Hills, IL, USA) and a high-voltage power supply (ZGF 60 kV, Shanghai Sute Corp., Shanghai, China) were used for coaxial electrospinning. All experiments were carried out under ambient conditions (24°C ± 2°C and relative humidity 57% ± 4%). The electrospinning process was recorded using a digital video recorder (PowerShot A490, Canon, Tokyo, Japan). After some initial optimization experiments, the applied voltage was fixed at 15 kV, and the nanofibers were collected on aluminum foil at a distance of 20 cm. All other parameters are listed in Table 1. The nanofibers obtained were dried for at least 24 h at 40°C under vacuum (320 Pa) in a DZF-6050 electric vacuum drying oven (Shanghai Laboratory Instrument Work Co. Ltd, Shanghai, China).

### Characterization

The morphology of the nanofiber mats was assessed using an S-4800 field emission scanning electron microscope (FESEM; Hitachi, Tokyo, Japan). Prior to examination, samples were platinum sputter-coated. The average nanofiber diameter was determined from at least 100 measurements in FESEM images, using the Image J software (National Institutes of Health, MD, USA). To observe the cross sections of the fibers, mats were placed into liquid nitrogen and manually broken prior to sputtering.

Transmission electron microscope (TEM) images of the samples were recorded on a JEM 2100 F field emission TEM (JEOL, Tokyo, Japan). Fiber samples were collected by fixing a lacey carbon-coated copper grid to the collector. X-ray diffraction (XRD) was conducted using a D/Max-BR diffractometer (Rigaku, Tokyo, Japan) over the 2θ range 5° to 60°. The instrument supplies Cu K*α* radiation generated at 40 mV and 30 mA. The raw quercetin particles were also studied under cross-polarized light using an XP-700 polarized optical microscope (Shanghai Changfang Optical Instrument Co. Ltd, Shanghai, China).

### *In vitro* dissolution tests

*In vitro* dissolution tests were carried out according to the Chinese Pharmacopoeia, 2005 ed. Method II, a paddle method, was performed using a RCZ-8A dissolution apparatus (Tianjin University Radio Factory, Tianjin, China). Drug-loaded nanofibers (200 mg) were placed in 900 mL of physiological saline (PS; 0.9 wt%) at 37°C ± 1°C. The instrument was set to stir at 50 rpm, providing sink conditions with *C* < 0.2*C*_s_. At predetermined time points, 5.0 mL aliquots were withdrawn from the dissolution medium and replaced with fresh medium to maintain a constant volume. After filtration through a 0.22-μm membrane (Merck-Millipore, Billerica, MA, USA) and appropriate dilution with PS, the samples were analyzed at *λ*_max_ = 371 nm using a UV/vis spectrophotometer (UV-2102PC, Unico Instrument Co. Ltd., Shanghai, China). Each experiment involved seven replicates: six of these were used to study drug release over a prolonged period of time. With the final replicate, the nanofiber mat was recovered after the first 5 min of dissolution and taken for further characterization.

## Results and discussion

### Coaxial electrospinning and the PVC-coated spinneret

**Figure 1:**
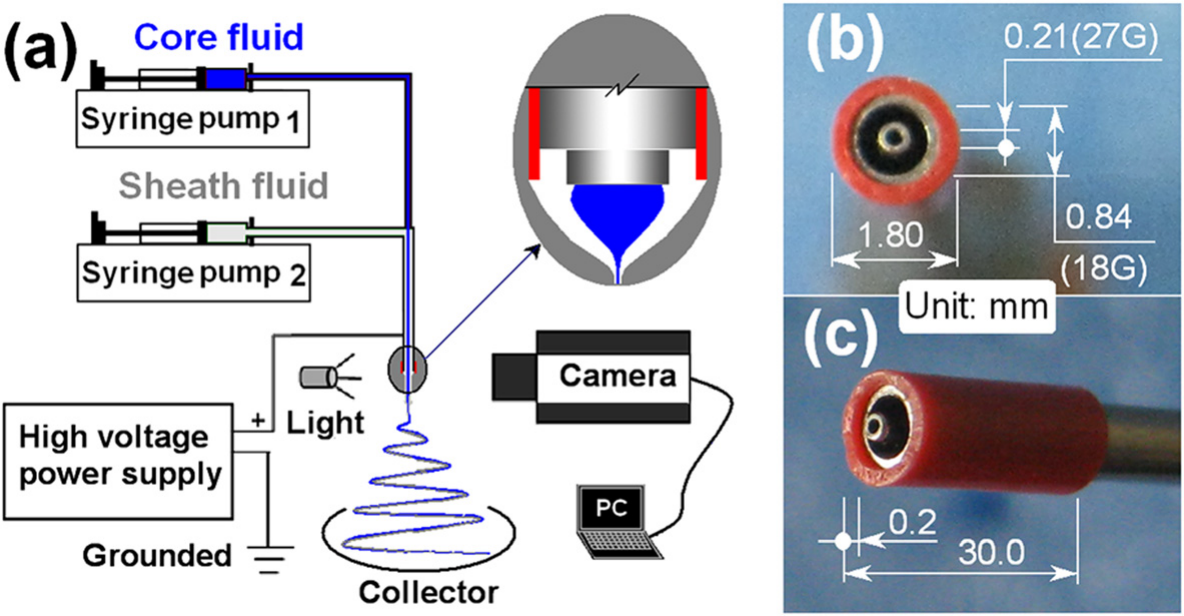
**The coaxial electrospinning apparatus and the PVC-coated concentric spinneret. (a)** A diagram of the coaxial electrospinning setup and **(b, c)** photographs of the PVC-coated concentric spinneret.

A schematic diagram of the coaxial electrospinning process is shown in Figure 1a. Photographs of the homemade PVC-coated concentric spinneret used are included in Figure 1b,c.

**Figure 2:**
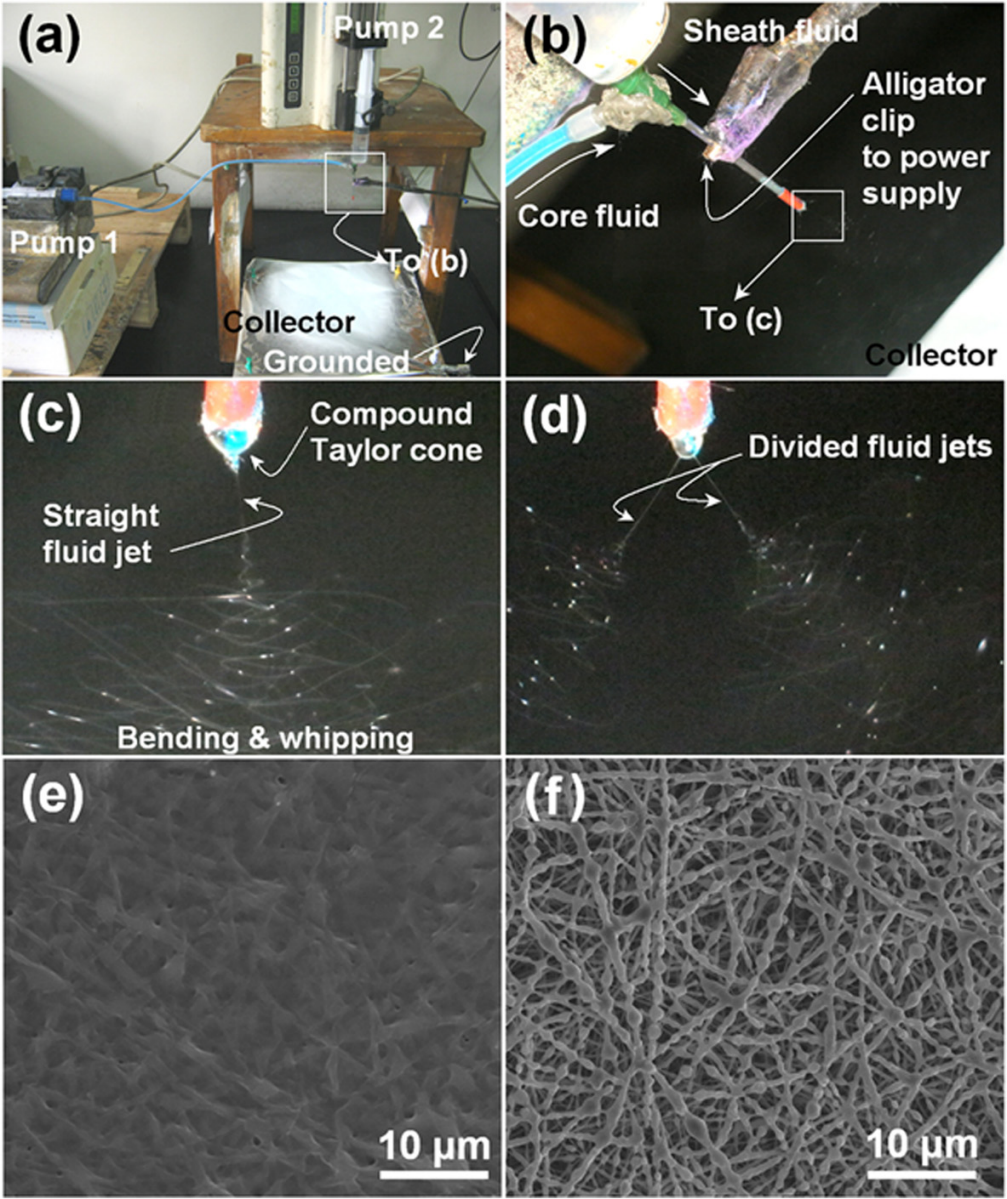
**Photographs of the coaxial electrospinning setup and the optimization of parameters. (a)** The apparatus used in this work, **(b)** the connection of the spinneret with the syringe pumps and power supply, **(c)** a typical coaxial process under an applied voltage of 15 kV with shell and core flow rates of 0.3 and 0.7 mL h^−1^, respectively, **(d)** the divided electrospinning process which was observed at 17 kV, **(e)** FESEM images of the F1 nanofibers resulting from single-fluid electrospinning of the shell fluid alone, and **(f)** FESEM images of fibers (F3) generated in a coaxial process with shell and core flow rates of 0.4 to 0.6 mL h^−1^, respectively.

When coaxial electrospinning was performed, two syringe pumps were used to drive the shell and core fluids independently (Figure 2a). An alligator clip was used to connect the metal part of the PVC-coated spinneret to the high-voltage power supply (Figure 2b). With an applied voltage of 15 kV and shell and core flow rates of 0.3 and 0.7 mL h^−1^, respectively, a successful electrospinning process was observed. A straight thinning jet was emitted from the compound Taylor cone and was then followed by a bending and whipping instability region with loops of increasing size (Figure 2c). Increasing the applied voltage to 17 kV resulted in a dividing of the straight fluid jet (Figure 2d). This complicated the process, increasing its instability and compromising the preparation of high quality of core-shell structures. Hence, the applied voltage was fixed at 15 kV.

For the preparation of drug-loaded nanofibers using a single-fluid electrospinning process, the selection of the solvent is often an important factor. It must meet three conditions: (i) the polymer should have good electrospinnability when dissolved in it, (ii) sufficient drug should dissolve in it to give a therapeutically useful drug content, and (iii) the resultant drug/polymer solution should be amenable to electrospinning. Hence, a mixed solvent is frequently used for generating monolithic drug-loaded nanofibers. The PVP shell matrix has good filament-forming properties in a wide variety of solvents such as ethanol, methanol, or chloroform. However, quercetin has poor solubility in all these solvents, instead dissolving easily in aprotic solvents such as dimethyl sulfoxide and DMAc. Unfortunately, PVP cannot be electrospun using these solvents because of their high boiling points. To balance these factors, a mixed solvent containing 30% DMAc and 70% ethanol was selected for the shell fluid. Although an electrospinning process could be observed when a voltage of 15 kV was applied to the shell fluid alone, solid nanofibers could not be obtained because the DMAc did not completely evaporate. After removal of the DMAc in a vacuum drying oven, a solid film was obtained, as depicted in Figure 2e. Thus, the shell fluid is deemed to be non-electrospinnable.

It is usually assumed that for coaxial electrospinning, the shell fluids must be electrospinnable [25,26]. However, our group has successfully developed a modified process, in which un-spinnable solutions can be used as shell fluids [14,15]. For these processes to proceed successfully, the shell-to-core flow rate ratio is a key parameter. Here, we found that a shell-to-core flow rate ratio of 2:3 (shell 0.4, core 0.6 mL h^−1^) resulted in an irregular morphology where numerous spindles and beads were visible along the nanofibers, as depicted in Figure 2f. To ameliorate this problem, a series of optimization experiments were performed. These led us to select shell and core flow rates of 0.3 and 0.7 mL h^−1^, respectively.

### The influence of PVC coating

**Figure 3:**
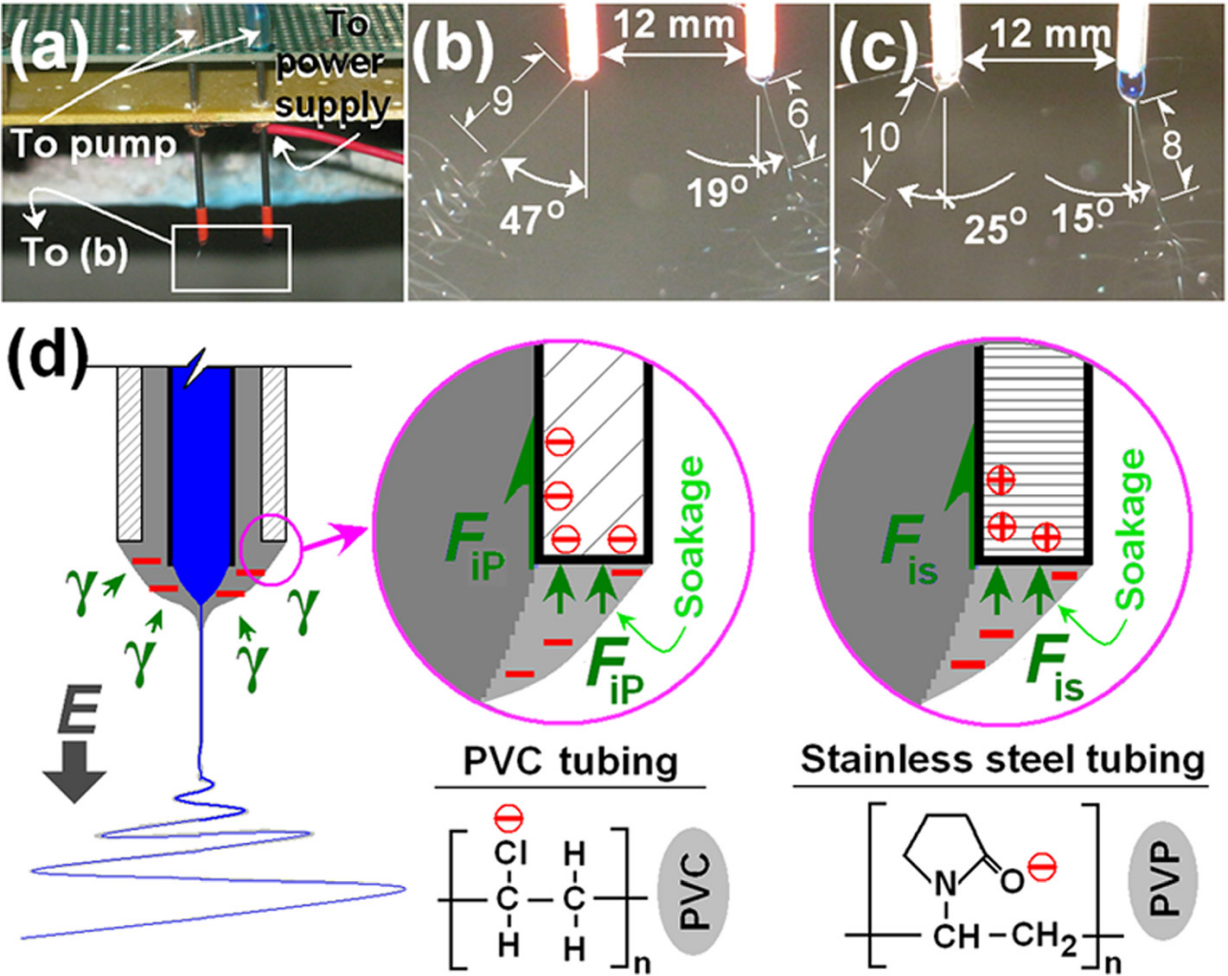
**Investigation of how the PVC-coated spinneret affects electrospinning. (a)** The experimental setup, **(b)** electrospinning with two PVC-coated spinnerets (inner diameter 1.0 mm), **(c)** spinning with two stainless steel spinnerets (inner diameter 1.0 mm), and **(d)** a schematic diagram illustrating the interfacial tensions between the sheath fluid and the spinneret. The sheath fluid is shown on the left and the core fluid on the right in **(b)** and **(c)**.

Based on our previous studies [27], it was expected that the PVC coating would lead to a more efficient electrospinning process. An experiment was designed to investigate this hypothesis, as shown in Figure 3a,b,c. Two separate spinnerets coated with PVC tubing (inner diameter 1.0 mm) were arranged in parallel at a distance of 12 mm apart. One was supplied with the shell fluid and the other with the core fluid. A typical image of the electrospinning process under an applied voltage of 15 kV and a flow rate of 1.0 mL h^−1^ is exhibited in Figure 3b. Similarly, two uncoated stainless steel spinnerets (inner diameter 1.0 mm) were arranged under the same conditions, and typical results are given in Figure 3c.

From a comparison of Figure 3b,c, a number of differences are clear: (i) when PVC-coated spinnerets were used, both fluids had a larger deflection angle than when the spinnerets were uncoated - for the shell fluid 47° > 25° and for the core 19° > 15°, (ii) the Taylor cones from the PVC-coated spinnerets are smaller than those from the metal spinneret, and (iii) the lengths of the straight fluid jets with the PVC-coated spinneret case are shorter than those using the metal spinneret, 9 mm < 10 mm (shell) and 6 mm < 8 mm (core). These results suggest that the PVC-coated spinneret conveys the electrical energy to the working fluids more effectively than the purely metal spinneret. This results in electrospinning commencing more rapidly with a smaller Taylor cone, shorter straight fluid jet, earlier onset of the instability region, and stronger repulsion forces between the two parallel fluids. Since it is an antistatic polymer, PVC can effectively retard the loss of electrical energy to the atmosphere.

The electrospinning process is governed by a balance between the surface tension of the liquid being spun and the electrical force. When the electrical forces are sufficiently large to overcome the fluid-restraining forces of surface tension, a Taylor cone is formed and a thinning straight jet emitted from it to initiate electrospinning. The literature emphasizes the influence of the surface tension between the liquid being processed and the atmosphere but overlooks the interfacial interactions between the working fluid and the inner wall of the spinneret. The latter must also play a key role in drawing the liquid back into the tube, thereby counteracting the electrical forces. Here, the interfacial tension between the shell fluid and PVC (*F*_iP_) should be lower than with a stainless steel nozzle (*F*_is_). This is expected to result from interactions with both the solvent and polymer solutes. A schematic is given in Figure 3d. A coaxial electrospinning process is traditionally deemed to a balance between the electrostatic field (*E*) and the surface tension of the shell fluid (*γ*). When a PVC-coated concentric spinneret is used, the abundant electron density of chlorine on the PVC surface causes it to repel the working solutions because of the electronegative oxygen atoms present in the PVP and EC molecules, suggesting a smaller interfacial tension. However, when a stainless steel spinneret is employed, the electropositive nature of the metal atoms makes them attract the shell solvent and solutes via their electronegative atoms. This not only increases the forces acting counter to the electrical drawing but also makes it easier for the electrospun fibers to become attached to the spinneret [27]. Thus, the PVC-coated spinneret can provide improved stability and impart increased robustness to the processes, producing higher quality nanostructures.

### Morphology and core-shell nanostructure

**Figure 4:**
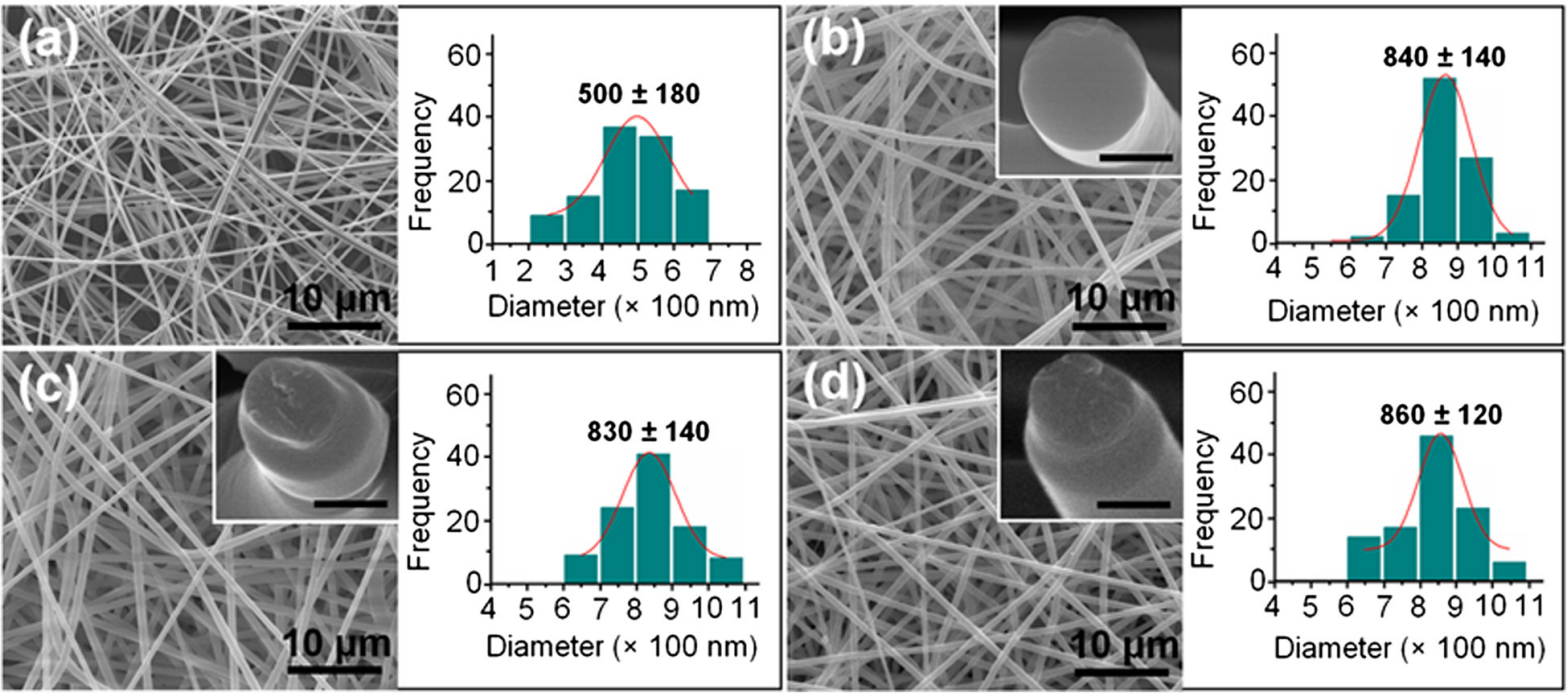
**FESEM images of the nanofibers and their diameter distributions. (a)** F2, **(b)** F4, **(c)** F5, and **(d)** F6. The scale bars in the insets of **(b, d)** represent 500 nm.

As shown in Figure 4, the quercetin-loaded fibers have smooth surfaces and uniform structures without any ‘beads-on-a-string’ morphology. The monolithic F2 fibers prepared through electrospinning only the core fluid had average diameters of 500 ± 180 nm (Table 1 and Figure 4a). The three core-shell nanofibers F4, F5, and F6 had average diameters of 840 ± 110 nm, 830 ± 140 nm and 860 ± 120 nm, respectively (Table 1 and Figure 4b,c,d). These results verify that high-quality nanofibers could be produced as a result of the electrospinnability of the core fluid, regardless of the inability to create solid materials from the shell solution alone via a single-fluid electrospinning.

FESEM images showing the cross-sections of the core-shell materials F4, F5, and F6 are given in the insets of Figure 4b,c,d. These cross-sections are smooth without discriminable nanoparticles generated by phase separation, which suggested a homogeneous structure in both the shell and core. A difference between F4 and F5/F6 is that the core-shell structures of the latter can be clearly seen in the projection of the core from the shell. This is thought to be associated with the increase of drug content, which makes the nanofibers brittle. The higher contents of quercetin in the shell of fibers F5 and F6 made them easier to fracture, and thus the core projects a little from the shell after breaking.

**Figure 5:**
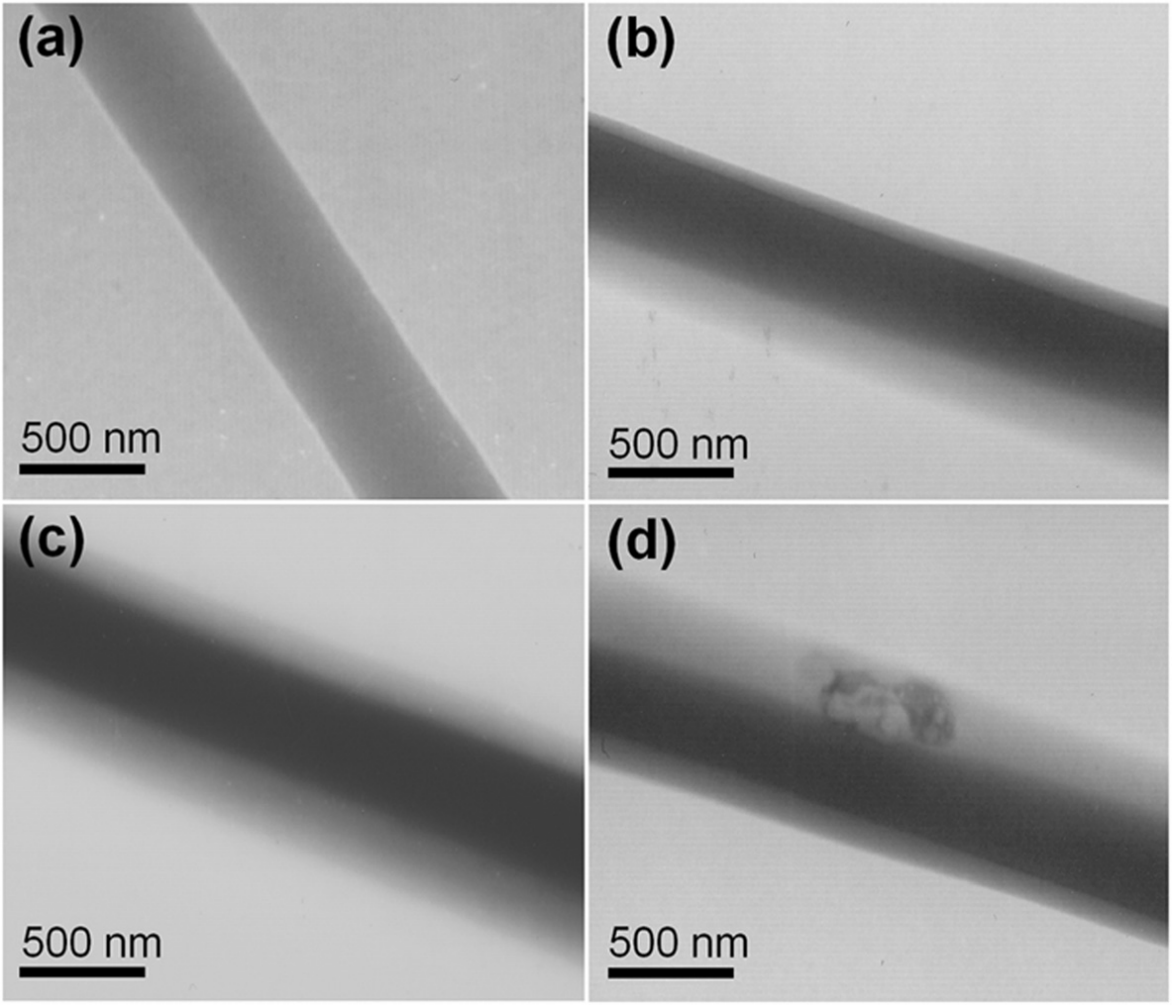
**TEM images. (a)** F2, **(b)** F4, **(c)** F5, and **(d)** F6.

TEM images of fibers F2, F4, F5, and F6 are shown in Figure 5. The uniform contrast of F2 suggests that the quercetin is distributed in the EC matrix at the molecular level, with no aggregates (Figure 5a). Fibers F4, F5, and F6 have evident core-shell structures (Figure 5b,c,d). Except for the heterogeneous region in the shell of F6 (see Figure 5d), no nanoparticles were observed in the three core-shell fibers, indicating uniform structures. The heterogeneous region in Figure 5d may be the result of a migration of the core components to the shell, or phase separation may have happened within the shell due to the high quercetin content in F6.

### Physical state of quercetin

**Figure 6:**
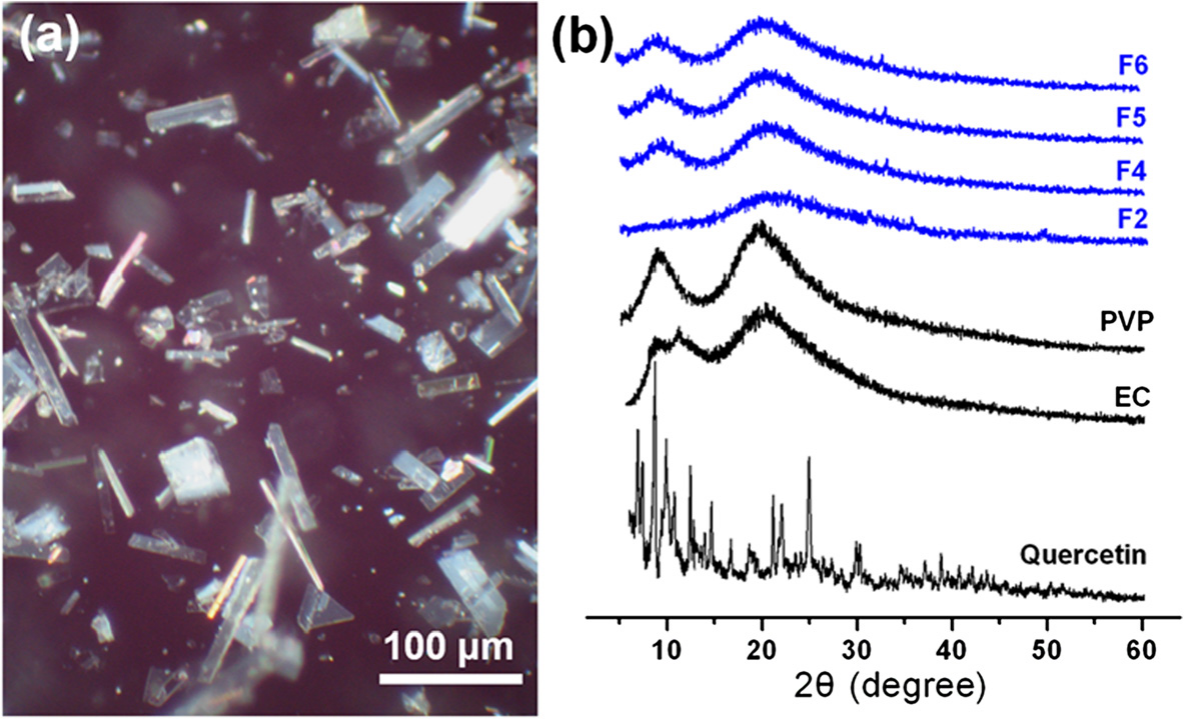
**Physical form investigation. (a)** Crystals of quercetin viewed under cross-polarized light and **(b)** XRD patterns of the raw materials and nanofibers.

XRD analyses were conducted to determine the physical status of the drug in the nanofibers. Quercetin, a yellowish green powder to the naked eye, comprises polychromatic crystals in the form of prisms or needles. The crystals exhibit a rough surface under cross-polarized light (Figure 6a). The data in Figure 6b show the presence of numerous distinct Bragg reflections in the XRD pattern of pure quercetin, demonstrating its existence as a crystalline material. The PVP and EC diffraction patterns exhibit a diffuse background with two diffraction haloes, showing that the polymers are amorphous. The patterns of fibers F2, F4, F5, and F6 show no Bragg reflections, instead consisting of diffuse haloes. Hence, the composite nanofibers are amorphous, and quercetin is not present as a crystalline material in the fibers.

These results concur with the SEM and TEM observations. No crystalline features are observed for any of the nanofibres. The heterogeneous region in Figure 5d is thus thought unlikely to be because of the recrystallization of quercetin, but most probably this anomaly comprises a composite of the drug and PVP with a higher concentration of quercetin than its surroundings.

### *In vitro* drug release profiles

**Figure 7:**
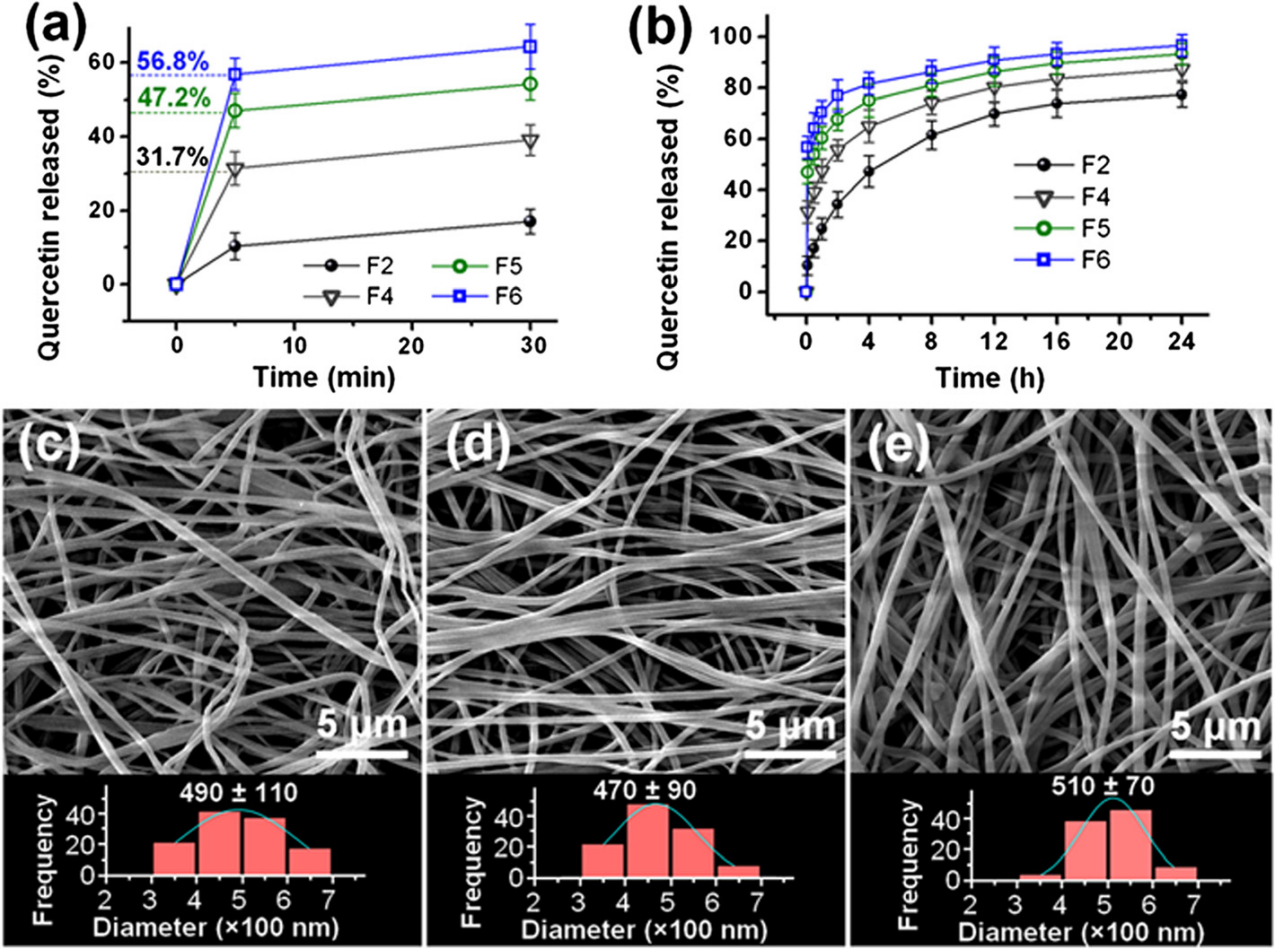
**In vitro drug release profiles.** Drug release **(a)** during the first 30 min and **(b)** over 24 h (*n* = 6), and FESEM images of the nanofibers after the initial stage of drug release: **(c)** F4, **(d)** F5, and **(e)** F6.

The *in vitro* drug release profiles of the four different nanofibers are given in Figure 7. As anticipated, the monolithic nanofibers F2 (containing only quercetin and EC) exhibited a sustained release profile as a result of the poor water solubility of quercetin and the insolubility of EC. In contrast, the core-shell fibers F4, F5, and F6 showed an initial burst release of 31.7%, 47.2%, and 56.8%, respectively, after they were placed in the dissolution medium for 5 min. These percentages are only very slightly larger than the calculated drug content in the shells of the fibers, suggesting that this initial burst release occurred almost solely from the fiber shells. This can be attributed to facts that (i) PVP is extremely hydrophilic, (ii) the fiber mats have very high surface areas and porosity, and (iii) electrospinning propagates the physical state of the components in the liquid solutions into the solid fibers to create homogeneous solid solutions or solid dispersions [28]. This means that despite being poorly soluble, the quercetin molecules can simultaneously dissolve with the PVP when the core-shell nanofibers are added to an aqueous medium, providing immediate drug release. After the first 5 min of rapid release, fibers F4, F5, and F6 exhibit sustained release with 87.5%, 93.4%, and 96.7% of the incorporated drug released after 24 h (Figure 7a,b).

Additional experiments were performed in which the fiber mats were recovered after 5 min in the dissolution medium and assessed by SEM. The recovered samples of F4, F5, and F6 were observed to have diameters of 490 ± 110 nm (Figure 7c), 470 ± 90 nm (Figure 7d), and 510 ± 70 nm (Figure 7e), respectively. This is around the same as the core diameters observed by TEM, indicating that the shell of the fibers had dissolved. The surfaces of the nanofibers remained smooth and uniform without any discernable nanoparticles, suggesting that quercetin in the shell was freed into the dissolution medium synchronously with the dissolution of the matrix PVP.

The quercetin release profiles from the EC nanofibers (F2) and the core of F4, F5, and F6 were analyzed using the Peppas equation [29]:

$$Q=kt^{n}$$

where *Q* is the drug release percentage, *t* is the release time, *k* is a constant reflecting the structural and geometric characteristics of the fibers, and *n* is an exponent that indicates the drug release mechanism. In all cases, the equation gives a good fit to the experimental data, with high correlation coefficients.

The results for F2 yield *Q*_2_ = 23.2 *t*_2_^0.42^ (*R*_2_ = 0.9855); an exponent value of 0.42 indicates that the drug release is controlled via a typical Fickian diffusion mechanism (this is the case when *n* < 0.45). For the cores of F4, F5, and F6, the regressed equations are *Q*_4_ = 13.7 *t*_*4*_^0.38^ (*R*_4_ = 0.9870), *Q*_5_ = 13.7 *t*_5_^0.36^ (*R*_5_ = 0.9866), and *Q*_6_ = 12.6 *t*_6_^0.31^ (*R*_6_ = 0.9881). These results demonstrate that the second phase of release from F4, F5, and F6 is also controlled by a typical Fickian diffusion mechanism.

Overall therefore, it is clear that tunable biphasic release profiles could be achieved from the core-shell nanofibers prepared in this work. The hydrophilic shell polymer provides fast initial release, and the core matrix subsequently furnishes sustained release.

## Conclusions

This study offers a simple approach for the systematic design and fabrication of biomaterials to provide complicated and programmable drug release profiles. A PVC-coated concentric spinneret was developed to conduct coaxial electrospinning, and quercetin-loaded core-shell nanofibers with tunable biphasic release profiles were fabricated. This could be achieved despite the fact that the shell fluid alone was found not to be electrospinnable. Electron microscopy demonstrated that the quercetin-loaded EC nanofibers and core-shell PVP/EC nanofibers had linear morphology and smooth surfaces. X-ray diffraction analyses indicated that the nanofibers contained quercetin in an amorphous physical form. *In vitro* dissolution tests showed that the fibers could provide biphasic release profiles consisting of initial fast and subsequent sustained release stages. The drug release in the latter phase occurred via a typical Fickian diffusion mechanism.

## Competing interests

The authors declare that they have no competing interests.

## Authors' contributions

D-GY and Z-HW conceived the idea of the project. CL and D-GY carried out the experiments. D-GY and GRW drafted the manuscript. GRW guided the revision of the manuscript. Z-HW supervised the project. All authors read and approved the final manuscript.
